# Reconstruction and Microstructure Characterization of Tailings Materials with Varying Particle Sizes

**DOI:** 10.3390/ma18163895

**Published:** 2025-08-20

**Authors:** Zhenkai Pan, Mingnan Xu, Tingting Liu, Junhong Huang, Xinping Li, Chao Zhang

**Affiliations:** 1 School of Civil Engineering and Architecture, Wuhan University of Technology, Wuhan 430070, China; panzhenkai23@whut.edu.cn (Z.P.); ttliu@whut.edu.cn (T.L.); junhonghuang@whut.edu.cn (J.H.); lixinping@whut.edu.cn (X.L.); 2 State Key Laboratory of Geotechnical Mechanics and Geotechnical Engineering, Institute of Rock and Soil Mechanics, Chinese Academy of Sciences, Wuhan 430071, China; 3 Key Laboratory of Mine Slope Safety Risk Warning and Disaster Prevention and Mitigation, Ministry of Emergency Management, Wuhan 430071, China

**Keywords:** tailings materials, particle size distribution, CT scanning technology, microstructure, 3D reconstruction

## Abstract

With the continuous increase in mining activities, effective tailings management has become a critical concern in geotechnical and environmental engineering. This study systematically investigates the microstructural characteristics and 3D reconstruction behavior of copper tailings with different particle sizes using X-ray computed tomography (micro-CT), digital image processing, and 3D modeling techniques. Two particle size groups (fine: 0.075–0.15 mm; coarse: 0.15–0.3 mm) were analyzed to quantify differences in particle morphology, pore structure, and orientation anisotropy. Binary images and reconstructed models revealed that coarse particles tend to have more irregular and angular shapes, while fine particles exhibit more complex pore networks with higher fractal dimensions. The apparent porosity derived from CT data was consistently lower than laboratory measurements, likely due to internal agglomeration effects. Orientation analysis indicated that particle alignment and anisotropy vary systematically with section angle relative to the principal stress direction. These findings offer new insights into the particle-scale mechanisms affecting the packing, porosity, and anisotropy of tailings, providing a scientific basis for enhancing the structural evaluation and sustainable management of tailings storage facilities.

## 1. Introduction

Granular materials are defined as substances characterized by discrete particulate constituents with loose properties. These materials are typically composed of a multitude of dispersed fine particles or particle aggregates, exhibiting a defined level of porosity. Common examples include soils, sands, gravels, pulverized coal, and various mineral powders [1,2,3]. The properties of granular materials are influenced by a variety of factors, including particle morphology, size distribution, bulk density, and porosity. Understanding these factors is essential for accurately predicting the mechanical behavior and performance of granular assemblies in both natural and engineered contexts. Tailings represent a form of waste generated during the mining process, primarily comprising metal and non-metal constituents, as well as potentially hazardous radioactive materials [4,5]. The effective management of tailings is crucial for environmental protection and the promotion of sustainable economic development. Addressing the challenges associated with tailings is essential for minimizing their ecological impact and enhancing the circular economy within the mining sector. Tailings materials are classified as granular materials due to their discrete, particulate structure and loose characteristics. Currently, the method most often used to manage tailings involves the in situ storage of these materials via the construction of engineered tailings dams. However, the failure of such dams poses significant risks, including substantial casualties, economic losses, and severe environmental degradation [6,7]. The mechanical properties of tailings are critical factors that influence the safety and stability of tailings reservoirs. The granular nature of tailings makes them susceptible to erosion by hydrodynamic forces, wind, and other environmental factors following accumulation, thereby posing risks related to environmental contamination and safety. The microstructure characteristics of tailings, including their particle morphology [8], anisotropy [9], and pore architecture [10], serve as essential parameters for characterizing the mechanical behavior of these materials. Therefore, investigating the micromechanical properties of tailings, such as particle contact forces, porosity, shear strength, and stiffness, is essential for understanding their stability and developing effective management strategies.

The microstructure of tailings materials is predominantly characterized using techniques such as optical microscopy, scanning electron microscopy (SEM), transmission electron microscopy (TEM), and atomic force microscopy (AFM) [11]. A considerable body of research has been dedicated to the microscopic characterization of geotechnical materials. Notably, Delage and Tessier [12] conducted extensive long-term studies on the microscopic structure of various geotechnical materials, identifying the fundamental principles and distinct microscopic mechanisms that govern the macroscopic volumetric changes in silt, bentonite, and clay. Hu et al. [13] investigated the effect of the movement of constituent particles in sandstone on the microscopic failure mechanisms of the material under varying loading rates, utilizing a compression testing system coupled with a microscope for real-time observation. Zhu et al. [14] employed atomic force microscopy (AFM) to investigate the nanostructure of hydrated calcium silicate (C-S-H), successfully obtaining a spatial distribution map that illustrates the tetrahedral arrangement of C-S-H nanoparticle aggregates. These methods can be employed not only to observe the surface morphology and color of the material but also to obtain high-resolution microscopic data, including information related to particle size, shape, density, and other structural characteristics. However, optical methods for characterizing surface texture are often limited by several factors, such as changes in the visual field, insufficient pore and particle information, and significant errors in characterizing microstructures, including particle shape and porosity. A modern alternative to optical techniques is the use of elastomeric sensors, which can effectively address these challenges. Elastomeric sensors are capable of documenting surface texture with higher accuracy by improving the acquisition of detailed surface features and overcoming issues like limited field-of-view. Recent studies, such as those by Peta et al. [15], have demonstrated the potential of elastomeric sensors in accurately capturing surface textures and microstructural features. These techniques involve substantial human intervention during sample preparation, which can lead to varying degrees of pore damage. These characterization methods are inherently two-dimensional, failing to capture the three-dimensional structural information of the samples. In order to accurately capture the three-dimensional structure of granular materials, it is essential to employ novel methods in order to reconstruct their microstructure. At present, the reconstruction techniques most commonly employed include morphological reconstruction [16], high-resolution computed tomography (CT) [17], and the digital scattering model (DSCM) [18]. However, research focusing on the reconstruction of the microstructure of tailings materials remains limited. The heterogeneous nature of the distribution of materials within tailings dams is influenced by variations in the emission concentration and discharge rate from individual tailings pipelines [19]. As a result, significant spatial heterogeneity exists in the particle size distribution across different regions of the dam. This variability poses considerable challenges in predicting the mechanical behavior of tailings materials and directly impacts the structural integrity and stability of the tailings dam. Consequently, it is crucial to develop and apply innovative techniques for characterizing and reconstructing the microstructure of tailings materials. In recent years, X-ray computed tomography (CT) has been extensively applied for microstructural characterization of geomaterials, including tailings and soils, due to its ability to provide high-resolution 3D images without destroying the sample [20,21]. Advanced segmentation and reconstruction techniques, such as watershed algorithms and machine learning-based methods, have further enhanced the accuracy of particle and pore structure quantification [22,23]. However, challenges remain in accurately extracting particle boundaries and quantifying mesostructural features, especially under conditions of complex particle–pore interactions.

This study investigates the effect of particle size on the characterization and reconstruction of the microstructure of tailings materials. CT scanning was performed on dried tailings samples with varying particle sizes. The resulting scan data were analyzed using advanced image processing techniques, enabling a comprehensive description of the internal microstructure of the tailings materials. The particle morphology of the tailings was characterized in terms of their shape and surface roughness. The distribution of the apparent porosity of tailings with varying particle sizes was examined, and the two-dimensional distribution of the pore size, pore fractality, and correlation between the apparent porosity and particle size were analyzed. The findings on particle packing, pore structure, and structural anisotropy can provide valuable insights into the mechanical behavior and potential failure mechanisms of tailings under varying stress conditions. These insights could aid in optimizing the material properties and stability predictions for tailings dams in real-world applications.

## 2. Materials and Methods

### 2.1. Tailings Materials

**Figure 1:**
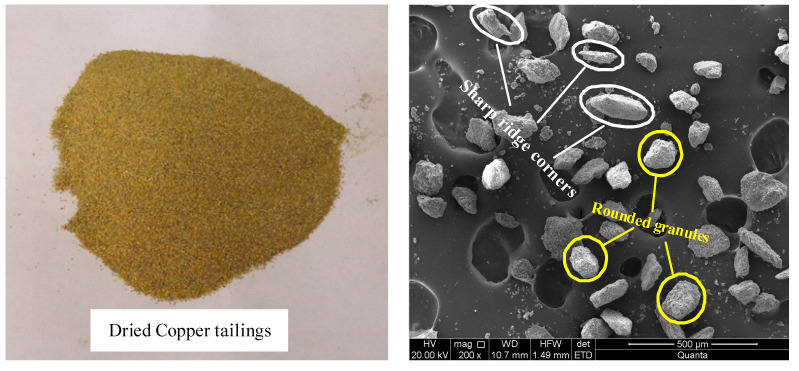
Original tailings and SEM images.

**Figure 2:**
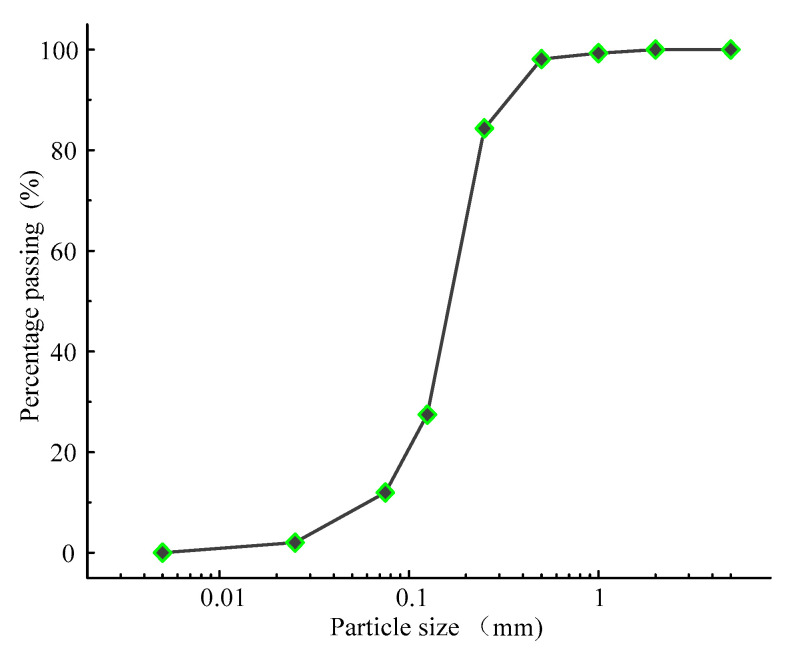
Particle size distribution for original tailings.

Tailings materials, sourced from a copper tailings reservoir in Jiangxi Province, exhibit a golden yellow color upon drying and are characterized by loose particle structures and a low degree of bonding between the tailings particles. To characterize the surface morphology, scanning electron microscopy (SEM) imaging was conducted using a JEOL JSM-IT500 microscope (Tokyo, Japan). Samples were sputter-coated with a thin layer of gold to enhance conductivity, and imaging was performed at an accelerating voltage of 15 kV and a magnification of 200×. As shown in Figure 1, the copper tailings particles display a wide variety of shapes, including angular, sharp-edged particles as well as more rounded and smoother forms. This variability in particle shape and surface texture suggests a heterogeneous distribution of particle sizes and forms within the tailings material. The original tailings sample exhibits an initial moisture content of 9.34%, a specific gravity of 2.76, and an initial porosity of 54.4%. The dry density of the tailings is 1.32 g/cm^3^. The particle size distribution curve for the tailings material is presented in Figure 2. The average particle size of the sample is 0.162 mm, with a uniformity coefficient (*C_u_*) of 3.9 and a curvature coefficient (*C_c_*) of 1.9. According to the Unified Soil Classification System (USCS) [24], the tailings are classified as having poor gradation.

### 2.2. Test Apparatus

**Figure 3:**
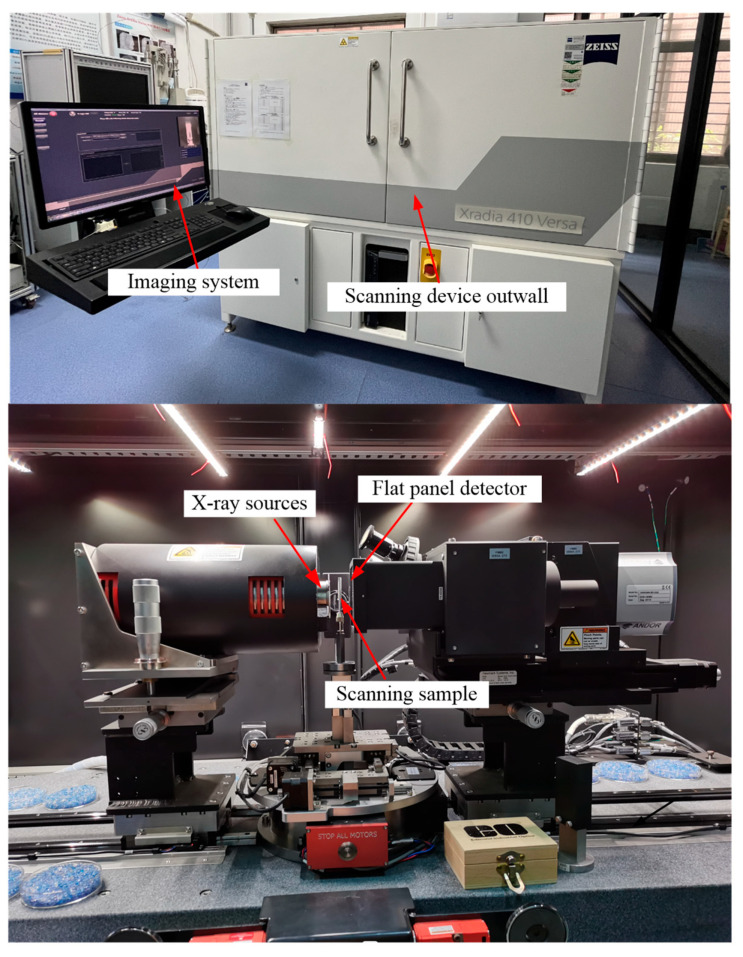
CT scanning imaging system and internal structure.

A high-performance micro-computed tomography (μCT) scanner (Zeiss Xradia 410 Versa, Jena, Germany) was utilized for the imaging analysis. Figure 3 illustrates the external contour of the scanning system as well as the configuration of the internal imaging components. This advanced imaging system enabled the high-resolution, non-destructive visualization of the sample’s internal microstructure. The device is composed of an internal closed X-ray source, a high-precision sample stage, an automated objective lens turntable, a detector (objective lens), a Macro-70 (0.4X) detector, a control system, and a computer workstation. The device operates with a maximum tube voltage of 160 kV and a maximum output power of 10 W. The sample stage supports a bearing capacity of up to 15 kg and is capable of 360° rotation, enabling multi-angle measurements. The system accommodates samples with a maximum allowable size of 30 cm, facilitating the analysis of larger specimens with high precision. During the scanning process, the sample is positioned on the sample stage, which rotates synchronously with the stage. The X-ray source, emitted from the left-side emitter, penetrates the sample as it rotates. The transmitted X-rays are subsequently processed by a scintillator, amplified, and captured using a fast CCD camera (Tokyo, Japan). The acquired data undergoes software-based processing to reconstruct a high-precision image of the sample’s internal structure, achieving an imaging resolution at the micron scale.

### 2.3. Test Preparation

**Figure 4:**
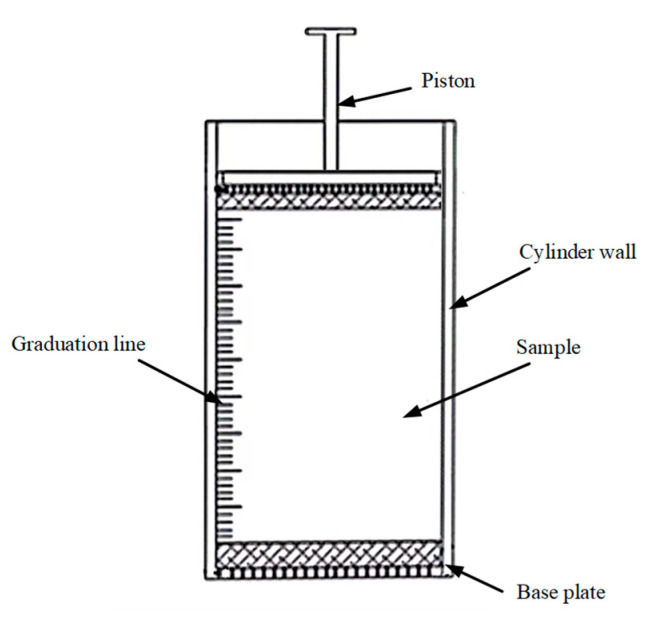
Schematic diagram of CT sample preparation device.

The test samples for the CT scans were prepared using a self-designed device, with its internal structure illustrated in Figure 4. CT scanning experiments were performed on two groups of tailings with different particle sizes, with the inner diameter of the scanning device controlled at 4 mm and the height maintained at 8 mm, ensuring that the samples had consistent dimensions for comparative analysis. The CT scanning parameters included a spatial resolution of 4.04 µm/pixel and a camera operating temperature of −59 °C. The spacing between tomographic slices was set to one pixel unit, and the slice image dimensions were 992 × 1014 pixels. A total of 998 eight-bit TIFF format CT images were acquired following the scan. The X-ray source was operated at 50 kV and 200 µA, with an exposure time of 2.5 s. The system employed an optical magnification of 4× and a camera readout speed of 2.5 MHz.

**Figure 5:**
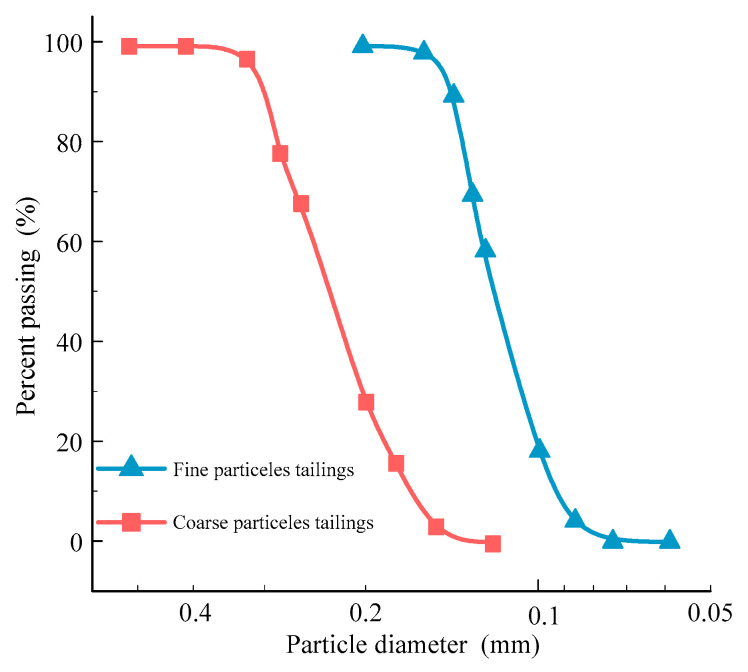
Particle size gradation curves of two different tailings sizes.

The sample preparation employed the sand rain technique, wherein the dried tailings were allowed to fall freely from a fixed height of approximately 30 cm into a cylindrical mold to ensure uniform deposition and minimize particle segregation. This process was repeated to prepare two tailings groups with distinct particle size distributions, which were subsequently used for CT scanning and further analysis. Figure 5 shows the particle size gradation curves of two groups of tailings with different particle sizes; tailings with particle sizes smaller than 0.075 mm were removed. The particle sizes of the two tailings groups were 0.075–0.15 mm (fine particles) and 0.15–0.3 mm (coarse particles), respectively. These ranges correspond to the dominant fractions observed in the particle size distribution of the tested tailings material, as determined by preliminary sieve analysis. The tailings samples were compressed by a piston, forming cylindrical specimens with an initial density of 1.62 g/cm^3^.

### 2.4. Digital Image Processing

Digital image processing involves the manipulation and analysis of digital image data stored in a computer following digital transformation [25]. A digital image consists of a series of pixels, each represented by a square unit. Digital image processing involves pixel-level operations performed on the image to extract, enhance, or interpret visual information. In a grayscale image, each pixel contains two types of information: the spatial coordinates of the pixel and its grayscale value. For an 8-bit grayscale image, the grayscale value of each pixel ranges from 0 to 255, representing 256 possible intensity levels, where 0 corresponds to black, 255 corresponds to white, and intermediate values represent varying shades of gray. In the binary image generated through numerical image processing, the pixel grayscale values are restricted to 0 and 1, where 0 represents a black pixel and 1 denotes a white pixel. The fundamental process of digital image processing primarily consists of enhancement processing, segmentation processing, and morphological image processing. The image undergoes gray histogram analysis, histogram equalization, and spatial domain filtering to extract the target image with improved clarity and contrast [26,27].

In this study, the publicly available image processing software ImageJ1.54, developed in Java by the National Institutes of Health (NIH) (Bethesda, MD, USA), was utilized to preprocess the original CT images and threshold segmentation. ImageJ is compatible with multiple operating systems, including Windows and Linux, and is widely applied in the field of image analysis. The software enables pixel counting, area measurement, and fractal dimension calculation, making it a versatile tool for quantitative image processing.

**Figure 6:**
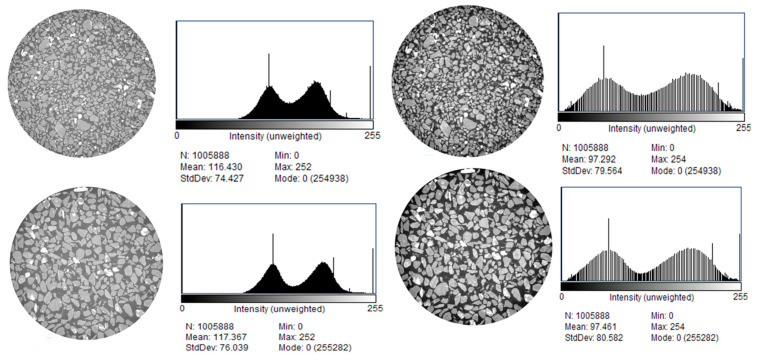
Images and histograms of 450th slice CT images before and after equalization.

Histogram equalization was applied to enhance the signal of the original image, improving its overall visual contrast and facilitating feature extraction and the analysis of target information. As shown in Figure 6, the gray value range of the fine- and coarse-grained tailings images is expanded after processing, aiding in the determination of the segmentation threshold. The equalized image also appears brighter and more effectively reveals the particle and pore regions compared to the original image.

**Figure 7:**
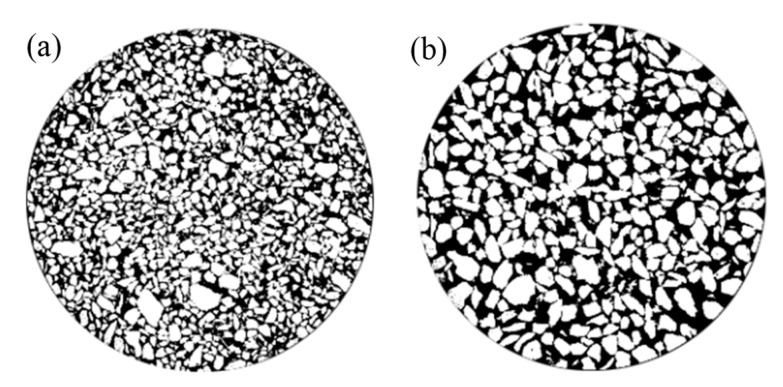
Binarization image of 450th slice CT scan images ((**a**) fine-grained tailings; (**b**) coarse-grained tailings).

The image pixels of the CT scan consist of pores and particles, which are segmented using an automatic thresholding method followed by binarization. In the binary image, black pixels (gray value = 0) represent tailings particles, while white pixels (gray value = 255) correspond to pores. The watershed segmentation method is applied to separate adjacent particles, providing a basis for the characterization of the microstructure of pores and particles. Figure 7 displays the 450th layer images of fine- and coarse-grained tailings after automatic threshold segmentation.

### 2.5. Three-Dimensional Reconstruction

CT technology enables the non-destructive acquisition of the internal microstructure of tailings, allowing particles and pores to be effectively distinguished through digital image processing [28]. However, the resulting two-dimensional (2D) transverse slices represent only cross-sectional profiles along the sample height and are insufficient for analyzing particle anisotropy. Therefore, the 3D reconstruction of 2D CT images is necessary to capture the complete spatial structure. CT technology enables the non-destructive acquisition of the internal microstructure of tailings, with particles and pores effectively identified through digital image processing. However, the acquired images are limited to two-dimensional (2D) transverse slices, which are insufficient for analyzing particle anisotropy. To address this, three-dimensional (3D) reconstruction of the CT images is performed, enabling a detailed investigation of the microstructure by extracting arbitrary cross-sections from the reconstructed 3D geometry.

**Figure 8:**
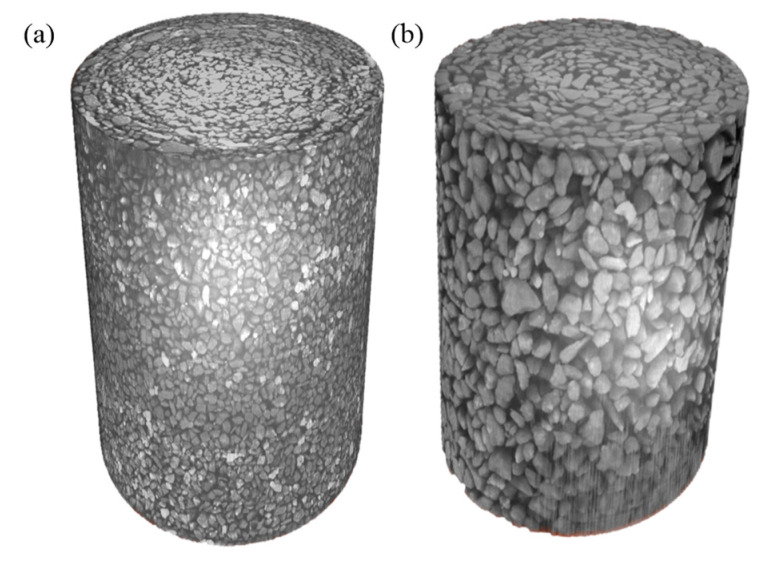
Three-dimensional reconstruction of tailings samples ((**a**) fine-grained tailings; (**b**) coarse tailings).

A total of 998 original CT images were batch-processed to obtain binarized images, which were then 3D-reconstructed using AVIZO 8.0 to achieve three-dimensional visualization. There are two commonly used reconstruction techniques, namely surface rendering and volume rendering [29]. This study adopts the volume rendering method. Volume rendering assigns distinct colors and transparency to different data types within the volume, reconstructing the 3D structure by projecting and restoring voxel attributes. Compared to surface rendering, volume rendering enhances quality and clarity of images, though it involves greater computational complexity. This method is selected to enable a more accurate analysis of the internal microstructure of tailings. The processed digital images were imported into AVIZO 8.0 for 3D reconstruction using the volume rendering method. Through 3D computation, the spatial geometric structures of fine- and coarse-grained tailings were obtained, as shown in Figure 8. Under uniform compression, fine-grained tailings exhibit smaller pores with a denser and more uniform distribution compared to coarse-grained tailings.

## 3. Effect of Particle Size on Microstructure Characterization

The microstructure of tailings, including their particle morphology and pore structure, fundamentally influences their macroscopic mechanical behavior. Traditionally, these features were analyzed using scanning electron microscopy (SEM), which involves preparing thin sections of soil samples for digital image analysis [30]. However, this approach is destructive and limited in its capacity to capture the spatial anisotropy of particles and the 3D pore structure, leading to significant methodological constraints and potential artifacts. To overcome the limitations of previous research on soil microstructure, this study utilizes micro-CT scanning, image processing, and 3D reconstruction to investigate the microstructure of tailings materials. Based on the reconstructed data, particle shape indices, spatial anisotropy, and pore distribution of tailings with varying particle sizes are statistically analyzed, providing a microscopic view of the morphological and structural characteristics of tailings.

### 3.1. Effect of Grain Size on Shape Parameters

**Figure 9:**
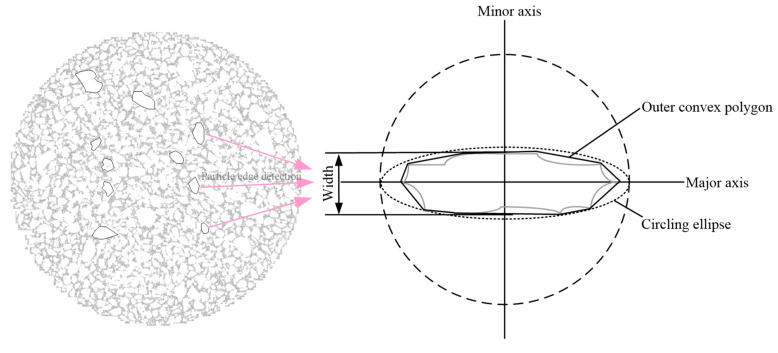
Particle edge detection and parameter extraction.

To improve the study of particle microstructure, the accurate extraction of individual particle information is essential. Although digital image processing effectively captures particle data, particle adhesion often complicates single-particle identification. This chapter addresses this issue by applying the watershed segmentation method in ImageJ to separate touching particles and extract boundary information [31]. Microscopic parameters such as the particle area (Area), boundary perimeter (Perimeter), and convex hull area (ConvexArea) are extracted from particle edges. A schematic of edge detection and parameter extraction is shown in Figure 9.

The appropriate calculation formulas are selected to determine the shape index of tailings particle, facilitating the study of particle microstructure. Two key indices, the shape factor (which characterizes particle shape) and solidity (which indicates the compactness or density of the particle), are chosen to evaluate the particle microstructure.

The shape factor is defined as the ratio of the particle boundary perimeter to the perimeter of an equivalent-area circle, as shown in Equation (1) [32].

(1) $$SF=\frac{P}{2\pi r}$$

where P is the perimeter of the particle, r is the equivalent radius, r=Aπ, and A refers to the particle area. The shape factor of the circle is 1.0; for an ellipse with a long-to-short axis ratio of 1.5:1, the shape factor is 1.08; for a ratio of 3:1, it is 1.31. A shape factor that deviates further from 1 indicates a greater departure from circularity in the particle’s shape.

Solidity is defined as the ratio of the particle area to the area of its convex polygon and is used to characterize the number of edges and corners on the particle surface, as well as the degree of convexity. The calculation formula is as follows:

(2) $$S=\frac{A}{A_{convex}}$$

where $A_{convex}$ is the area of the convex polygon of the particle. A solidity value closer to 1 indicates a smoother particle surface.

**Figure 10:**
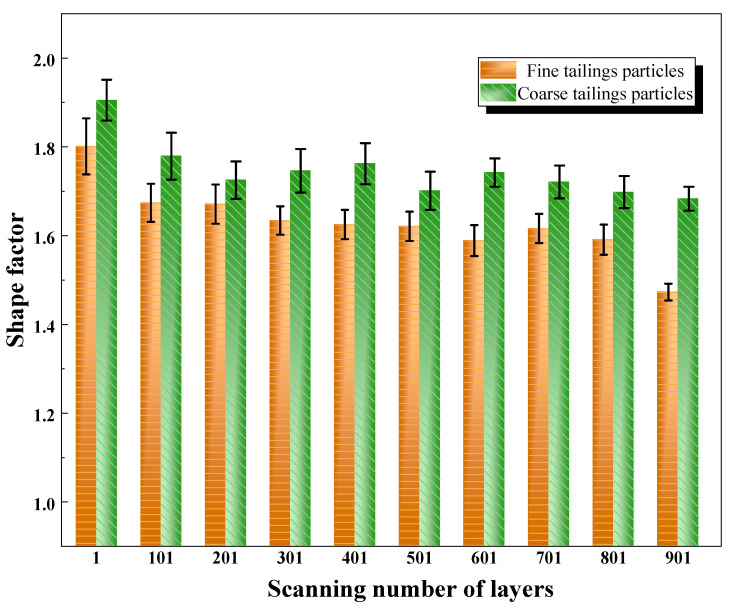
Variation in shape factor with scanning layer number.

The shape indices of tailings particles in CT scan images processed using the watershed segmentation method can be quantitatively calculated; this includes the average shape factor and solidity within the scanned section. The variation in the shape factor of tailings particles with the number of CT scanning layers (i.e., sample height) is shown in Figure 10. The results indicate that the shape factor of both fine- and coarse-grained tailings decreases as the scanning depth increases. This trend is primarily attributed to the deposition process in tailings: the weight and stress exerted by upper particles promote denser packing in lower layers. As depth increases, the influence of overlying stress diminishes, leading to a more uniform arrangement of particles, reduced interparticle interaction, and an increase in the ease of particle reorientation, thereby resulting in a lower shape factor. At all stacking positions, coarse particles exhibit higher shape factors than fine particles. Additionally, the shape factors for both coarse- and fine-grained tailings exceed 1.4, indicating a significant deviation from circularity. The two-dimensional morphology reveals elongated and flattened characteristics, with a long-to-short axis ratio greater than 3:1.

**Figure 11:**
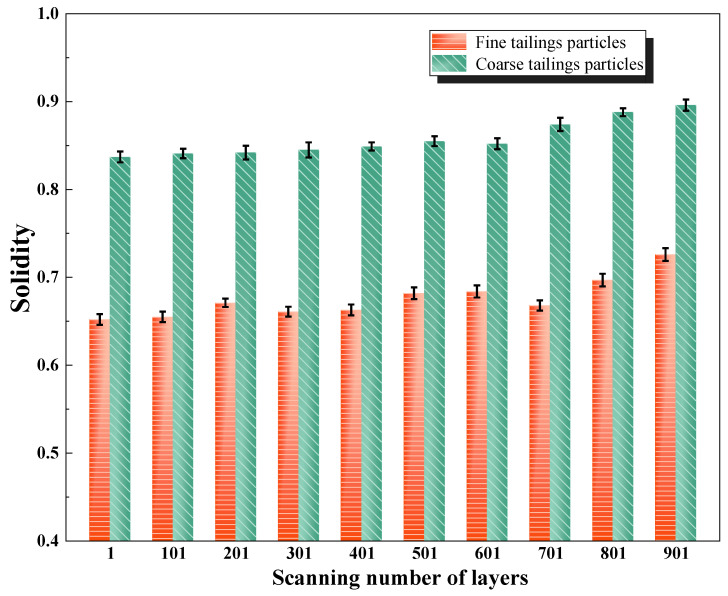
Variation in solidity with scanning layer number.

As shown in Figure 11, the solidity of tailings particles varies with the number of scanning layers. Since the solidity values are all below 1, the particle surfaces are highly irregular, exhibiting significant roughness and angularity. Smaller particles display lower solidity values, indicating rougher and more angular surfaces. This may result from the transport process, where larger particles bear more external load and undergo mutual abrasion, leading to smoother surfaces, while smaller particles, shielded within the voids, experience less friction and retain sharper edges [33]. As the scanning depth increases, the solidity of particles increases, primarily due to compaction in the lower layers of the tailings. As a result, particles become more densely and orderly arranged, reducing surface irregularities and leading to smoother particle morphologies.

### 3.2. Effect of Grain Size on Porosity Ratio

The spatial pore characteristics of tailings are typically quantified by porosity. In CT binary images, each pixel represents a square unit, with particle pixels assigned a value of ‘1’ (white) and pore pixels ‘0’ (black). The apparent porosity of a single CT slice is calculated as the ratio of pore pixels to the total number of pixels.

**Figure 12:**
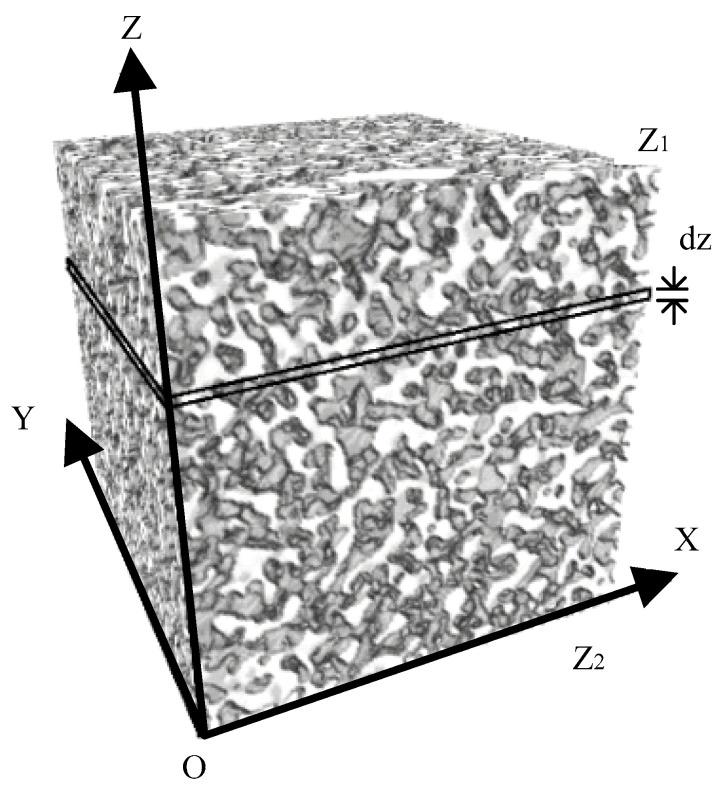
Computational model.

By statistically analyzing the apparent porosity of a series of binary CT images, both the vertical distribution of porosity along the sample height and the overall volume porosity can be determined. Based on the method in the literature [34], a computational model is established as shown in Figure 12. Let $d_{z}$ be the CT slice spacing, $z_{1}$ and $z_{2}$ be the upper and lower boundaries, $n_{z}\left(i\right)$ be the apparent porosity at height z, and i be the slice index from the top to bottom. When $d_{z}$ is sufficiently small, the volume porosity between two adjacent slices at height z can be expressed as follows:

(3) $${\bar{n}}_{z}=n_{z}\left(i\right)$$

Then, the volume porosity of the computational model can be obtained:

(4) $$\bar{n}=\left(\sum_{i=1}^{k}n_{z}\left(i\right)\right)/k$$

where k represents the total number of $d_{z}$ intervals between $z_{1}$ and $z_{2}$.

**Figure 13:**
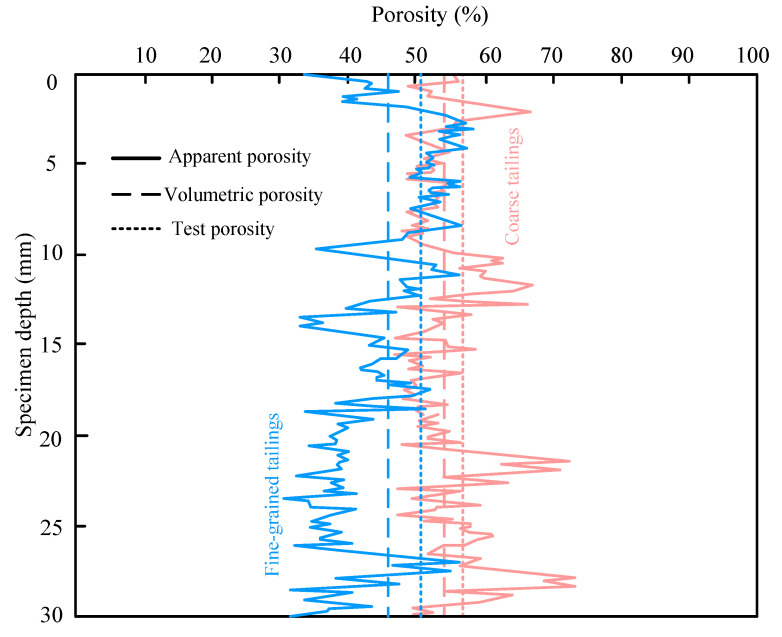
Tailings porosity distribution.

By applying Equations (3) and (4) in conjunction with the total sample height, the volume porosity of the entire specimen can be determined. Figure 13 presents the spatial distribution curves of both the volume porosity and apparent porosity along the sample height, as calculated from the binary CT images of fine- and coarse-grained tailings. The porosity of fine- and coarse-grained tailings, measured using standard sieving and moisture methods, is 50.12% and 56.60%, respectively. Under identical initial density and specific gravity, fine particles exhibit denser packing and a reduced pore space. In contrast, the larger size and higher shape factor of coarse particles result in fewer contact points and increased pore volume. These differences arise from the distinct particle arrangements and void structures formed during deposition. The porosity of fine- and coarse-grained tailings calculated from the 3D reconstruction of binary images is 46.68% and 53.79%, respectively. Both are lower than values obtained through laboratory testing. Laboratory methods, such as the volume or saturation method, may be affected by particle agglomeration, reducing measurement accuracy. The corresponding error rates are 6.86% for fine-grained and 4.96% for coarse-grained tailings. These small deviations indicate that the computed porosity is sufficiently accurate to represent the actual porosity. The apparent porosity of fine-grained tailings along the specimen fluctuates between 33.15% and 46.77%, while that of coarse-grained tailings ranges from 45.67% to 71.12%. A frequency analysis of porosity values across CT slices shows that fine-grained tailings exhibit a bimodal distribution, primarily concentrated in the ranges of 35–40% and 48–52%. In contrast, coarse-grained tailings display a symmetric distribution centered around 45–55%, with a decreasing frequency beyond 57%. This bimodal pattern in fine-grained tailings is attributed to the presence of clay minerals, which promote aggregation and result in both intra-aggregate and inter-aggregate pores.

### 3.3. Effect of Grain Size on Pore Size Distribution

**Figure 14:**
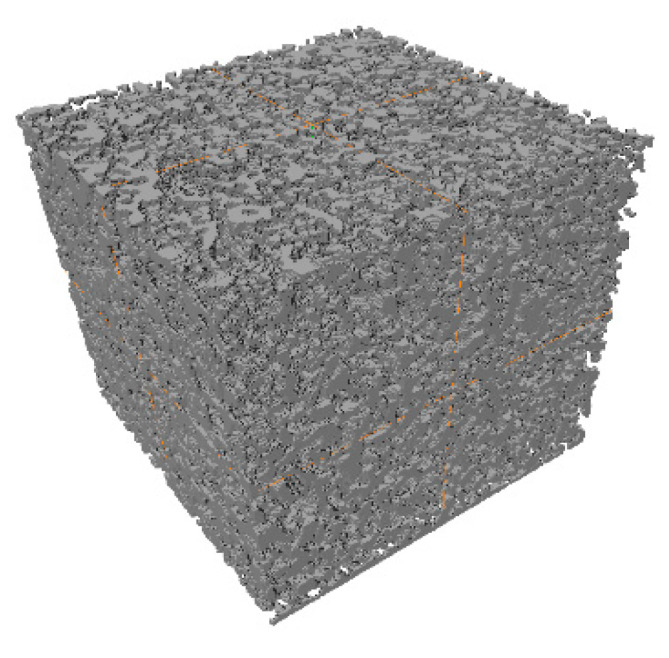
Pore network model.

The internal pore size and its spatial distribution are critical factors influencing the mechanical strength characteristics of soil. In this study, a quantitative analysis of pore size and distribution was conducted based on two-dimensional binary images using MATLAB 2023 programming. The pore size at each point along the pore skeleton was calculated to obtain a comprehensive pore size distribution profile. To minimize the impact of scanning-induced artifacts, statistical analysis was confined to a centrally located rectangular region within the sample, thereby ensuring the accuracy and reliability of the results. The reconstructed pore network model based on two-dimensional images is presented in Figure 14, and the corresponding quantitative results are summarized in Table 1. For coarse-grained tailings, the pore size ranges from 30 μm to 360 μm, with the majority of pores being smaller than 100 μm, followed by those in the 100–200 μm range; pores larger than 200 μm account for the smallest proportion. Fine-grained tailings exhibit a similar distribution pattern, with pore sizes ranging from 17 μm to 285 μm, but show a notably higher proportion of pores smaller than 100 μm. The higher content of cementitious and organic matter in fine-grained tailings results in a generally smaller pore size. The pore size distribution is negatively correlated with particle size. Therefore, changes in particle size lead to variations in the pore structure. These changes affect the physical properties of soil, such as its permeability, aeration, and water-holding capacity, thereby influencing its ecological functions and environmental impact [35].

### 3.4. Effect of Grain Size on Pore Fractal Characterization

In recent years, fractal theory has been increasingly used to investigate the pore structures of rocks, coal, and other geotechnical materials, yielding significant research advancements. A fundamental characteristic of fractal theory is the self-similarity inherent to the structures under analysis. Xie [36] was the first to introduce fractal theory into the study of rock microstructures, demonstrating that the fracture networks within rocks exhibit pronounced fractal behavior. In this study, the pore structure of tailings is selected as the primary subject of investigation, and fractal theory is employed to quantitatively characterize its geometric complexity. The box-counting method, in conjunction with ImageJ software, is utilized to calculate the fractal dimension based on binary images of the pore structure.

The box-counting dimension is defined as follows:

Let F be a non-empty, bounded subset of $R^{n}$, and let $N\left(\varepsilon \right)$ denote the number of boxes of side length ε required to entirely cover the set F. The upper and lower box-counting dimensions of F are then given by the following:

(5) $$\underline{\dim_{B}}F=\underset{\sigma \to 0^{+}}{\lim}\frac{InN\left(\varepsilon \right)}{-In\varepsilon }$$

(6) $$\bar{\dim_{B}}F=\underset{\sigma \to 0^{-}}{\lim}\frac{In\left(\varepsilon \right)}{-In\varepsilon }$$

If Equations (5) and (6) are equal, the shared value is referred to as the box-counting dimension of the set F. In this case, the box dimension of F is expressed as follows:

(7) $$\dim_{B}F=\underset{\sigma \to 0}{\lim}\frac{InN\left(\varepsilon \right)}{In\varepsilon }$$

where $N\left(\varepsilon \right)$ denotes the minimum number of boxes with side length ε required to completely cover the set F. This dimension serves as a quantitative indicator of the fractal complexity inherent in the spatial structure of F.

In the fractal box-counting method, a digital image is treated as a surface, Z=f(x,y), in a two-dimensional space, where f(x,y) denotes the pixel coordinate and represents the gray value at the corresponding point. For binary images, the gray value Z is restricted to either 0 or 1. Given an image of size M×M, a series of square boxes with side length ε are used to cover the set of pore pixels (i.e., pixels with a grayscale value of 0). The number of boxes N(ε) required to completely cover the pore regions is recorded at each scale. According to the definition of fractal dimension, a linear relationship is expected between logN(ε) and logε. The slope of the line fitted to the set of data points $\left(\log\varepsilon_{i},\log N(\varepsilon_{i})\right)$, where *i* = 1,2,…,*k*, is determined using the least squares regression method. The fractal dimension D of the image surface is then obtained as the negative value of the slope of this fitted line.

**Figure 15:**
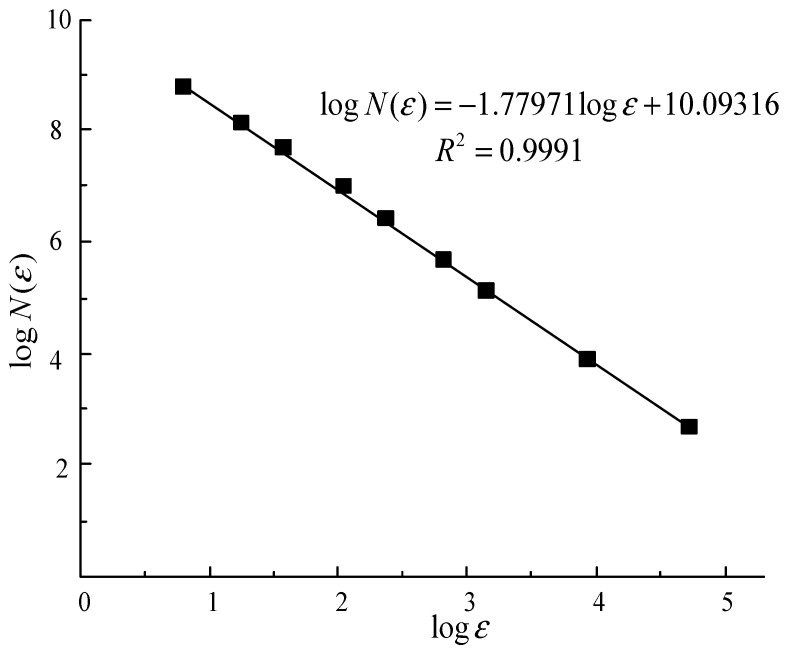
Double logarithmic plot of box size against number of boxes for tailings pores.

A total of 50 binarized images were extracted along the vertical axis of the sample. Based on Equation (5), the relationship between box size and the corresponding number of boxes was analyzed using the fractal box-counting method implemented in ImageJ software. The data were plotted in a double logarithmic coordinate system. As illustrated in Figure 15, the fractal dimension D of the pore structures on the cut surfaces ranges from 1.689 to 1.787, with a coefficient of determination $R^{2}$ varying between 0.9974 and 0.9994. These results indicate a strong linear correlation and suggest that the pore structures within the tailings exhibit pronounced self-similarity and self-organizing characteristics.

**Figure 16:**
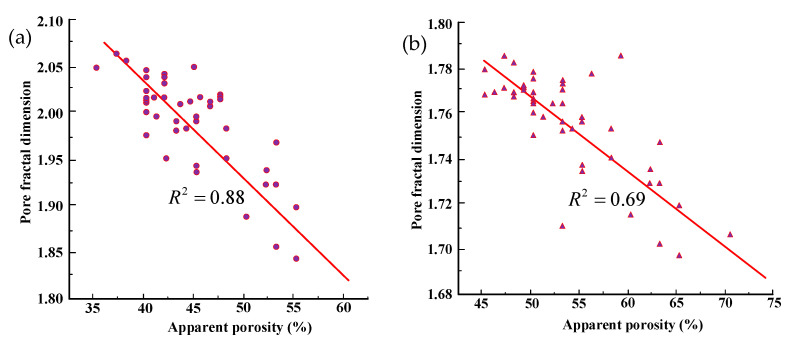
Relation between pore fractal dimension and apparent porosity: (**a**) fine-grained tailings; (**b**) coarse-grained tailings.

The pore fractal dimension and corresponding apparent porosity of each CT slice were statistically analyzed to investigate their relationship. As shown in Figure 16, the pore fractal dimension generally decreases as the apparent porosity increases, though the decline is relatively gradual. For coarse tailings, the apparent porosity increased from 46.5% to 70.6% (an increase of 51.83%), while the fractal dimension decreased from 1.787 to 1.689 (a decrease of 5.48%). In fine tailings, the apparent porosity rose from 35.26% to 55.68% (an increase of 57.91%), with the fractal dimension decreasing from 2.062 to 1.846 (a decrease of 10.48%). The results indicate that the pore fractal dimension decreases only slightly with increasing apparent porosity, showing a negative correlation. The relationships for fine- and coarse-grained tailings can be expressed as follows:

(8) $$D_{f}=2.44-1.058n$$

(9) $$D_{c}=1.98-0.407n$$

where n is the apparent porosity, and $D_{f}$ and $D_{c}$ are the pore fractal dimensions of fine- and coarse-grained tailings, respectively.

The pore structure of fine-grained tailings exhibits greater complexity compared to that of coarse-grained tailings. At the same level of apparent porosity, fine-grained tailings show higher fractal dimensions, reflecting a more intricate pore structure and a more heterogeneous pore distribution. The pore fractal dimension of fine-grained tailings is more sensitive to changes in apparent porosity compared to that of coarse-grained tailings. When the porosity approaches zero, the fractal dimensions are 2.44 and 1.98 for fine- and coarse-grained tailings, respectively. This difference may be attributed to variations in the mineral composition and processing methods between the two materials [37].

## 4. Statistical Evaluation of Anisotropy Based on Particle Orientation in Tailings

**Figure 17:**
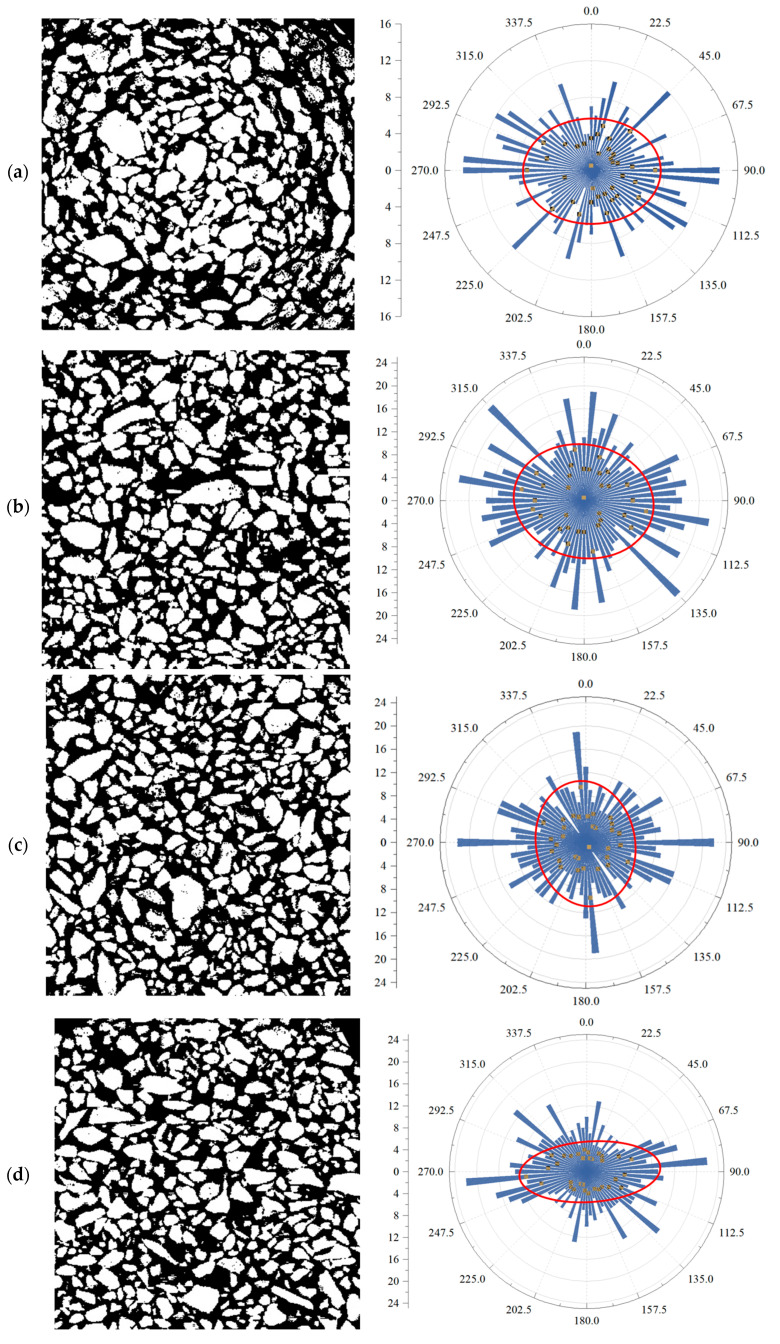
Binarized images and corresponding particle orientation rose diagrams for different sections: (**a**) maximum principal stress section; (**b**) 30° section; (**c**) 45° section; (**d**) 60° section.

Directivity statistical analysis of particles is a quantitative method used to assess the particle spatial distribution, orientation, and arrangement. It can be employed in particle engineering and soil and rock mechanics [38]. Particle orientation is a key micromechanical indicator of soil structure. In this study, the main orientation angle, rose diagram, and anisotropy index are employed to characterize the orientation features of the tailings samples. Figure 17a–d show binarized images of representative tailings sections and the corresponding particle orientation rose diagrams and fitted ellipses (taking coarse-grained tailings as an example). The rose diagrams are constructed based on the orientation angles of individual particles, with an angular interval of 5°, where the length of each sector represents the number of particles within that orientation range.

Since particle orientation angles are defined within the range of 0°–180° and exhibit directional symmetry, the rose diagram over 0°–360° is obtained by mirroring the 0°–180° data. An optimal fitting ellipse is generated from the full dataset, with the angle of its major axis representing the main orientation of particles in the section. Furthermore, the anisotropy ratio of particle orientation can be calculated based on the fitted ellipse parameters [39] as follows:

(10) $$I=\frac{a-b}{a}$$

where I denotes the particle anisotropy ratio in the binary image, while a and b represent the lengths of the major and minor axes of the fitted ellipse, respectively.

The orientation indices of each section are summarized in Table 2. On the maximum principal stress plane, the main particle orientation angle is close to 90°, with the lowest anisotropy ratio among all sections. As the section angle increases to 30°, the main orientation angle increases slightly, while the anisotropy remains relatively unchanged. At 45°, the main orientation angle continues to rise, approaching 180°, accompanied by a slight increase in anisotropy. At 60°, the main orientation angle decreases, but the anisotropy reaches its maximum, indicating that the orientation angle first increases and then decreases, while the anisotropy ratio increases consistently (within the 0°–60° range). This suggests that the 60° section exhibits the most pronounced particle alignment and structural anisotropy, consistent with the commonly observed shear band inclination of 45°–75° during shear failure.

## 5. Conclusions

This study systematically investigates the microstructural characteristics and reconstruction behavior of tailings materials with varying particle sizes, contributing new insights into the field of tailings characterization. The following key conclusions, highlighting both methodological advancements and novel findings, can be drawn:

(1) Binary images of particles and pores were obtained through advanced CT scan data processing, including filtering, binarization, and the watershed segmentation method. This methodological innovation enabled the precise calculation of particle shape indices, with a focus on particle elongation and angularity, providing a more comprehensive understanding of tailings microstructure. Notably, the shape factor of coarse-grained tailings is higher than that of fine-grained tailings, and particle solidity decreases with decreasing particle size, reflecting compactness or density variations, which have not been fully addressed in previous studies.
(2) This study introduces a 3D reconstruction model for calculating porosity, revealing that fine-grained tailings exhibit denser packing and more complex pore structures, which significantly affect their mechanical and transport properties. The study also explores the fractal dimension of tailings pore structure, finding that fine-grained tailings have higher fractal dimensions under the same apparent porosity, suggesting greater complexity in their pore networks compared to coarse-grained tailings. These findings provide a novel insight into the relationship between particle size, porosity, and pore structure in tailings materials.
(3) A novel statistical analysis of particle orientation based on binary images reveals significant anisotropy in the particle alignment at different section angles (principal stress surface, 30°, 45°, 60°, and 90°). The study shows that structural anisotropy is the most pronounced at a 60° angle relative to the principal stress, providing a deeper understanding of the microstructural behavior of tailings under varying stress conditions. This approach offers a new perspective on how the orientation of particles influences the material properties and can aid in more accurate predictive modeling for engineering applications involving tailings.

## Figures and Tables

**Table 1:** Pore size distribution.

Sample	Max Pore Size (μm)	Min Pore Size (μm)	2D Pore Size Distribution (%)		
			<100 μm	100~200 μm	>200 μm
Coarse-grained tailings	360	30	56.82	39.67	3.51
Fine-grained tailings	285	17	70.23	27.32	2.45

**Table 2:** Orientation indices of each section.

Cross-Section	Major Axis of Fitting Ellipse	Minor Axis of Fitting Ellipse	Main Orientation Angle	Anisotropy Ratio (%)
Max principal stress	14.5	11.8	81.56	18.62
30°	24.3	19.7	97.88	18.93
45°	23.1	16.2	166.51	29.87
60°	25.7	11.2	84.38	56.42

## Data Availability

The original contributions presented in this study are included in the article. Further inquiries can be directed to the corresponding author.
